# Tuberculosis in prison inmates in Southern Brazil: investigating the epidemiological and operational indicators

**DOI:** 10.1590/0037-8682-0052-2022

**Published:** 2022-10-24

**Authors:** Caroline Busatto, Julia Mespaque, Pauline Schwarzbold, Camilo Darsie de Souza, Carla Adriane Jarczewski, Rodrigo Dalke Meucci, Jason Andrews, Julio Croda, Pedro Eduardo Almeida da Silva, Ivy Bastos Ramis, Lia Gonçalves Possuelo

**Affiliations:** 1 Universidade Federal do Rio Grande, Programa de Pós-Graduação em Ciências da Saúde, Rio Grande, RS, Brasil.; 2 Superintendência dos Serviços Penitenciários, 8ª Delegacia Penitenciária Regional, Santa Cruz do Sul, RS, Brasil.; 3 Universidade de Santa Cruz do Sul, Programa de Pós-Graduação em Educação, Santa Cruz do Sul, RS, Brasil.; 4 Secretaria Estadual da Saúde, Programa Estadual de Controle da Tuberculose, Porto Alegre, RS, Brasil.; 5 Stanford University, School of Medicine, Stanford, California, USA.; 6 Universidade Federal de Mato Grosso do Sul, Faculdade de Medicina, Campo Grande, MS, Brasil.; 7 Universidade de Santa Cruz do Sul, Programa de Pós-Graduação em Promoção da Saúde, Santa Cruz do Sul, RS, Brasil.

**Keywords:** Epidemiology, Monitoring, Tuberculosis, Public health, Prisons

## Abstract

**Background:**

Tuberculosis is a worldwide public health problem and is more prevalent in specific populations, such as prisoners. The aim of this study was to analyze the epidemiological and operational indicators of tuberculosis in prisoners in a southern region of Brazil.

**Methods:**

This was a descriptive, observational study, utilizing secondary data from the Notifiable Diseases Information System on tuberculosis cases diagnosed in prisoners in the state of Rio Grande do Sul, southern Brazil, from 2014 to 2018. Prisoner data used to calculate incidence were extracted from reports by the National Penitentiary Department.

**Results:**

From 2014 to 2018, 3,557 tuberculosis cases were reported in Rio Grande do Sul prisoners. The incidence rate of tuberculosis in prisoners was 1,235/100,000 individuals in 2014 and 1,430/100,000 individuals in 2018. The proportion of new TB cases tested for HIV was high, 83.4% in this period; among those tested, 12.9% were HIV coinfected. The proportion of new cases of pulmonary tuberculosis confirmed by laboratory criteria was 52.6% in this period. In total, 18.4% of new pulmonary tuberculosis cases were initiated on directly observed treatment in this period, and 36.4% of contacts of new cases of pulmonary tuberculosis with laboratory confirmation were examined. Among retreatment pulmonary tuberculosis cases, 82.4% were laboratory-confirmed.

**Conclusions:**

Tuberculosis incidence is increasing on a per-capita and absolute basis in Rio Grande do Sul. Laboratory confirmation, HIV testing, directly observed treatment, and contact investigation rates were all low, indicating the need to improve medical and public health measures for tuberculosis control in prisons.

## INTRODUCTION

Tuberculosis (TB) is among the leading infectious causes of mortality worldwide, causing the death of 1.4 million people annually^1^. Brazil, with approximately 90,000 new cases reported annually, is among the 30 World Health Organization-designated high burden countries^2^. As in many countries, incidence rates of TB are related to comorbidities and sociodemographic factors, and consequently, some populations, such as prisoners, are more likely to develop the disease. Accordingly, the incidence in Brazil is approximately 35 times higher than in the general population^3^ ^,^ ^4^.

The prison environment contributes to the spread of *Mycobacterium tuberculosis*, as well as the development of TB, due to poor ventilation, limited exposure to sunlight, overcrowding, smoking, drug and alcohol use, and in many facilities, insufficient healthcare resources. In addition, there is a constant movement of prisoners between facilities, increasing the possibility of dissemination of *M. tuberculosis* throughout prison systems^5^ ^,^ ^6^. 

The current prison population in Brazil is approximately 750,000 people, almost twice (171%) the prison capacity of the country. In Rio Grande do Sul (RS), over 41,000 individuals are incarcerated and capacity is at 168%^7^. In recent years, the proportion of TB cases occurring among prisoners nationally has increased substantially, and incarcerated individuals now comprise one of the largest TB risk groups in the country^2^ ^,^ ^8^.

Knowledge about the epidemiological and operational indicators of TB is fundamental for the planning of interventions to control the disease, by identifying areas for interventions and systems improvement^9^. Therefore, this study aimed to analyze the epidemiological and operational indicators of TB in prisoners in a state in southern Brazil.

## METHODS

This was a descriptive, observational study that included TB cases diagnosed in prisoners in RS from 2014 to 2018, reported in the Notifiable Diseases Information System (SINAN). SINAN is a national information system predominantly consisting of cases of diseases and conditions that appear on the national list of compulsory notifications. Access to the secondary database of SINAN was granted by the Health Secretary of RS on December 18, 2020. Individuals without incarceration status reported at the time of TB diagnosis and under 18 years old were excluded. 

**FIGURE 1:**
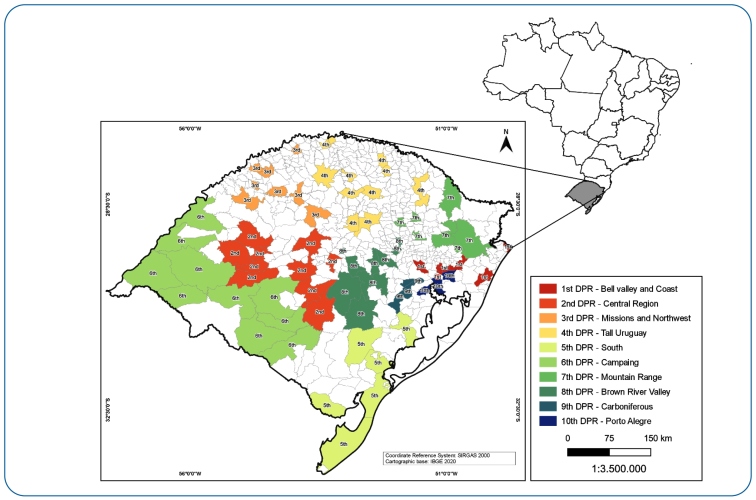
Regional Penitentiary Delegacy (DPR) of Rio Grande do Sul state, Brazil^17^.

The state of RS, located in southern Brazil, has approximately 42,000 prisoners, distributed in 105 closed-regime prisons. These prisons are distributed in ten Regional Penitentiary Delegacies (DPR) throughout RS, as shown in Figure 1. RS has the fifth largest prison population in Brazil, with an incarceration rate of 363/100,000 inhabitants^7^.

Following the recommendations of the Brazil Ministry of Health^9^, the incidence was calculated by DPR and by year, and operational indicators were calculated by year (Table 1). Prisoner data used to calculate the incidence coefficient were extracted from reports by the National Penitentiary Department^7^. 

**TABLE 1:** Description of tuberculosis epidemiological and operational indicators^9^.

Name	Indicator Description
	**Epidemiological**
**TB Incidence coefficient**	Number of new cases^a^ of TB/total prison population x 100,000
	**Operational**
**Proportion of TB/HIV coinfection among new TB cases**	Number of new cases^a^ of TB testing positive for HIV/total number of new cases^a^ of TB x 100
**Proportion of HIV testing among new TB cases**	Number of new cases^a^ of TB that underwent HIV testing (positive, negative, in progress)/number of new cases^a^ of TB x 100
**Proportion of antiretroviral therapy in total new cases with TB/HIV coinfection**	Number of new cases^a^ with TB/HIV coinfection undergoing antiretroviral therapy/new cases^a^ with TB/HIV coinfection x 100
**Proportion of new cases of pulmonary TB confirmed by laboratory criteria**	Number of new cases^a^ of pulmonary TB with confirmation by laboratory criteria^b^/total number of diagnosed TB cases x 100
**Proportion of cure among new cases of pulmonary TB with laboratory confirmation**	Number of cures among new cases^a^ of pulmonary TB confirmed by laboratory criteria^b^/new pulmonary cases confirmed by laboratory criteria^b^ x 100
**Proportion of treatment abandonment among new cases of pulmonary TB with laboratory confirmation**	Number of treatment abandonment among new cases^a^ of pulmonary TB confirmed by laboratory criteria^b^/new cases^a^ of pulmonary TB confirmed by laboratory criteria^b^ x 100
**Proportion of new cases of pulmonary TB that underwent directly observed treatment**	Number of new cases^a^ of pulmonary TB that underwent directly observed treatment/total new cases^a^ of TB x 100
**Proportion of examined contacts of new cases of pulmonary TB with laboratory confirmation**	Number of examined contacts of new cases^a^ of pulmonary TB confirmed by laboratory criteria^b^/new cases of pulmonary TB confirmed by laboratory criteria^b^ x 100
**Proportion of cases of pulmonary TB retreatment** ^c^ **confirmed by laboratory criteria**	Number of pulmonary TB retreatment^c^ cases with laboratory criteria^b^/total pulmonary cases of retreatment^c^ x 100
**Proportion of cure among laboratory-confirmed pulmonary TB retreatment cases**	Number of cures among pulmonary TB retreatment^c^ cases confirmed by laboratory criteria^b^/pulmonary TB retreatment^c^ cases confirmed by laboratory criteria^b^ x 100
**Proportion of treatment abandonment among laboratory-confirmed pulmonary TB retreatment cases**	Number of abandonment of pulmonary TB retreatment^c^ cases confirmed by laboratory criteria^b^/pulmonary TB retreatment^c^ cases confirmed by laboratory criteria^b^ x 100

a New cases: people with TB registered in SINAN as a new case, unknown and post-death.

b Laboratory criteria: People with TB who have had at least one positive result on laboratory tests (sputum smear microscopy, rapid molecular test, or sputum culture).

c Retreatment: TB cases registered in SINAN as re-entry after abandonment and recurrence.

Descriptive analyses, with absolute numbers and proportions calculations, were performed using Microsoft Office Excel^®^ (Micosoft Corporation, 2016, Redmond, WA, USA) and the Statistical Package for the Social Sciences (SPSS, version 20.0). The study was approved by the Institutional Review Board (IRB) of the School of Public Health and the State Health Secretary of RS.

The ‘Pi’ program (QGIS, version 3.14.15)^10^ was used for the preparation of thematic maps. The vector bases of the urban area, the municipalities, and the DPR were collected in the geoprocessing sector of the Municipality of Santa Cruz do Sul, RS^11^. These data were entered into the QGIS software, where all thematic and geoprocessing maps were edited. The colors with the warmest shades represented the highest incidence rates of cases. The final layout of the maps was also developed using QGIS. The maps were produced using the Coordinate Reference System (SRC), SIRGAS2000, which is the official standard in Brazil.

## RESULTS

**FIGURE 2:**
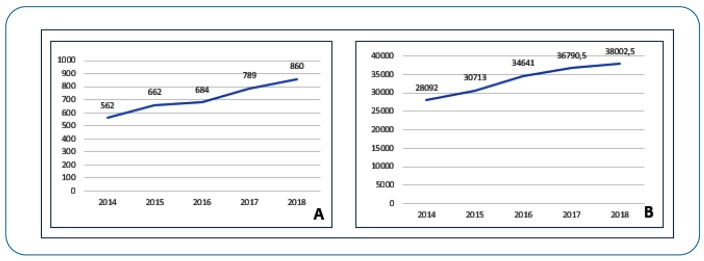
**(A)** Total tuberculosis cases in prisoners of Rio Grande do Sul per year; **(B)** Total prisoners in Rio Grande do Sul per year.

From 2014 to 2018, 31,511 cases of TB were reported in RS, of which 3,557 (11.3%) occurred in prisoners, increasing by 53% from 562 cases in 2014 to 860 cases in 2018 (Figure 2).

**FIGURE 3:**
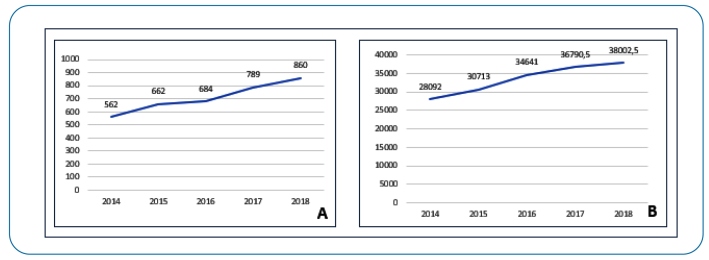
Incidence by Regional Penitentiary Delegacy (DPR).

The incidence of TB in prisoners was 1,235/100,000 individuals in 2014 and 1,429/100,000 individuals in 2018 (Table 2). Incidence ranged substantially by DPR, from 557/100,000 to 2,117/100,000 (Figure 3).

**TABLE 2:** Epidemiological and operational indicators of TB in prisoners in Rio Grande do Sul, 2014-2018.

Indicators	2014		2015		2016		2017		2018		Total	
TB incidence coefficient	1,235	-	1,338	-	1,213	-	1,465	-	1,429	-	1,369	-
	n/N	%	n/N	%	n/N	%	n/N	%	n/N	%	n/N	%
**TB/HIV** **co-infection among new TB cases**	49/347	14.1	72/411	17.5	56/409	13.7	51/530	9.6	64/566	11.3	292/2,263	12.9
**HIV testing among new TB cases**	262 ∕ 347	75.5	362∕ 411	88.0	364∕ 409	88.9	430∕ 530	81.1	470 ∕ 566	83.0	1,888∕ 2,263	83.4
**ART in total new cases with TB/HIV co-infection**	5/49	10.2	26/72	36.1	26/56	46.4	16/51	31.4	25/64	39.0	98/292	33.6
**New cases of pulmonary TB confirmed by laboratory criteria**	279/562	49.6	347/662	52.4	337/684	49.2	452/789	57.2	458/860	53.2	1,873/3,557	52.6
**Cure among new cases of pulmonary TB with laboratory confirmation**	172/279	61.6	223/347	64.3	226/337	67.0	292/452	64.6	275/458	60.0	1,188/1,873	63.4
**Treatment abandonment among new cases of pulmonary TB with laboratory confirmation**	64/279	22.9	52/347	14.9	40/337	11.9	36/452	7.9	35/458	7.6	227/1,873	12.1
New cases of pulmonary TB that underwent DOT	96/347	27.6	71/411	17.2	61/409	14.9	92/530	17.3	97/566	17.1	417/2,263	18.4
**Examined contacts of new cases of pulmonary TB with laboratory confirmation**	76/279	27.2	101/347	29.1	141/337	41.8	184/452	40.7	180/458	39.3	682/1,873	36.4
Cases of pulmonary TB retreatment* confirmed by laboratory criteria	127/160	79.4	165/189	87.3	143/176	81.2	145/178	81.5	158/192	82.3	738/895	82.4
**Cure among laboratory-confirmed pulmonary TB retreatment cases**	61/127	48.0	95/165	57.6	85/143	59.4	91/145	62.7	80/158	50.6	412/738	55.8
**Treatment abandonment among laboratory-confirmed pulmonary TB retreatment cases**	34/127	26.7	26/165	15.7	28/143	19.5	20/145	13.7	12/158	7.5	120/738	16.3

**n:** absolute number, **N:** denominator, **TB:** tuberculosis, **HIV:** human immunodeficiency virus, **ART:** antiretroviral therapy, **DOT:** directly observed treatment.

The main operational indicators are described in Table 2. The proportion of new TB cases tested for HIV was 83.4% in this period, while the proportion of TB/HIV co-infection among new TB cases was 12.9% and antiretroviral therapy (ART) in total new cases with TB/HIV co-infection was 10.2% in 2014, reaching 46.4% in 2016, with a rate of 33.6% overall. The proportion of new cases of pulmonary TB confirmed by laboratory criteria was 52.6% in this period. The curative rate among new cases of pulmonary TB with laboratory confirmation was 63.4%; while the proportion of treatment abandonment among new cases of pulmonary TB decreased from 22.9% in 2014 to 7.6% in 2018.

The examined contacts of new cases of pulmonary TB with laboratory confirmation was 36.4% in this period. There was an average of 82.4% cases of pulmonary TB retreatment confirmed by laboratory criteria during this period. Of the new cases of pulmonary TB in prisoners, 18.4% were treated under directly observed therapy (DOT), decreasing from 27.6% in 2014 to 17.1% in 2018. The proportion of cases cured increased from 48.0% in 2014 to 62.7% in 2017, while treatment abandonment among laboratory-confirmed pulmonary TB retreatment cases declined from 26.7% in 2014 to 7.5% in 2018. 

## CONCLUSIONS

From 2014 to 2018, we observed a 53% increase in TB reporting among prisoners in RS. While incidence rates rose by 15%, the majority of this increase was due to rising incarceration rates. During this period, prisoners comprised 11.3% of TB cases in the state. A similar rate was found in Brazil overall, which has shown an increase in TB cases among prisoners in the last 10 years (6.4% in 2010 and 11.1% in 2019)^2^. These rates represent the number of TB cases among this vulnerable population, emphasizing the importance of interventions to control the disease in this population and the general community^12^ ^,^ ^13^.

The incidence of TB in prisoners increased during the period studied, especially from 2017 onwards. This was the year in which the Rapid Molecular Testing Network for TB expanded with the distribution of equipment throughout the country, an advance achieved through the implementation of the Brazilian National Plan to End Tuberculosis as a Public Health Problem. In addition, in the Brazilian population in general, there was also a constant decreasing trend in incidence between the years 2010 and 2016, which reversed between 2016 and 2018^2^ ^,^ ^13^ ^-^ ^15^.

The 10th DPR corresponds to the metropolitan region of RS, which, despite being smaller in terms of territory, had a greater number of prisoners and a higher average incidence. It is considered a priority area and therefore has been availed more resources to care for the disease. Notably, the capital of RS, Porto Alegre, is located in this penitentiary region, where many cases are reported and referred for investigation of symptoms, diagnosis, and initiation of the therapeutic regimen. Patients with more complex clinical conditions are often kept in hospital until they are stable enough to continue the TB treatment in their original penitentiary region^16^ ^,^ ^17^.

People living with HIV in Brazil are estimated to be at 21 times greater risk of developing active TB than the general population^4^. In Brazil, TB/HIV coinfection in new cases of TB is decreasing in the general population, reaching 8.8% in 2018, but the southern region is still responsible for the highest proportions of TB/HIV coinfection (13.9% in RS)^2^. Although still higher than the national rate, a decrease in the TB/HIV coinfection in prisoners was observed in 2017-2018, compared with 2014-2016. The high level of HIV testing in prisoners, with an average of 83.4%, has almost reached the Ministry of Health's goal of testing all prisoners diagnosed with TB for HIV infection. Additionally, we observed that among the total new TB/HIV coinfection cases, the number of prisoners who underwent ART increased from 10.2% in 2014 to 39.0% in 2018. This emphasizes the importance of HIV testing in TB patients as this rate directly influences the access to and the correct implementation of ART ^5^ ^,^ ^18^.

The other operational indicators selected for this study were part of a list defined by the Brazil Ministry of Health for the periodic evaluation of TB control interventions. We observed an increase in the proportion of new cases of pulmonary TB confirmed by laboratory criteria when we compared 2014-2016 with 2017-2018. The rate of pulmonary TB retreatment found during the study period was significant (82.4%). Therefore, incarceration offers an opportunity for the diagnosis and treatment of TB and other infections in this vulnerable population, which often has insufficient access to health services outside of prison^9^ ^,^ ^13^.

Among new cases of TB, the maximum cure rate for pulmonary cases confirmed by laboratory criteria was 67.0% in 2016, while the maximum cure rate among pulmonary TB retreatment cases was 62.7% in 2017. However, cases of treatment abandonment considerably decreased over the period (approximately 67%). These indicators do not meet the goals set by the National Plan to End Tuberculosis, which aims for an outcome of 85% for the cure of the disease and less than 5% for treatment abandonment. Likewise, the Ministry of Health recommends that more than 70% of contacts of new cases of pulmonary TB with laboratory confirmation be examined. However, the average proportion of this indicator found in the period of our study was 36.4%, and rates lower than 50% are considered inadequate^5^ ^,^ ^16^. Therefore, considering the characteristics of the disease and the prison context (such as frequent transfers between prisons and prisons without health facilities), this aim becomes more difficult to achieve, given the existence of operational difficulties in health services regarding the management of TB^19^ ^,^ ^20^. 

The proportion of new cases of pulmonary TB in prisoners that underwent DOT was higher in 2014 (27.6%). This could be due to the implementation of the Prison Primary Care Team in 2014, according to the National Policy for Comprehensive Health Care for People Deprived of Liberty in the Prison System where the main objective is access to comprehensive health care. There are currently 45 health teams in closed prison units in RS, which represents coverage of 54.5% of inmates^21^. Since then, there has been an improvement in prisoners’ access to health services, including TB diagnosis and DOT, which is a valuable strategy toward greater adherence to treatment. However, the prison environment presents some difficulties for carrying out DOT, such as the mobility of prisoners within the prison, resulting in the health team often not having daily access to a prisoner, which may have impacted the DOT rate in the following years with an average of 16.6% between 2015 and 2018^22^ ^,^ ^23^.

The limitations of this study included low treatment completion and/or underreporting, due to the shortcomings of a study done using secondary data. However, the use of this data is important in the evaluation of health problems and in the planning of health policies and implementations that reflect the legitimate needs of the population, especially in the case of prisoners.

The indicators analyzed in this study showed the persistence of TB among prisoners of RS and reflected the limitations in the monitoring of prisoners with TB, indicating the need to develop systematic implementations aimed at this vulnerable population. Therefore, the importance of health surveillance in the monitoring of TB cases in prisoners and the strengthening of primary health care within prisons is reinforced. We believe that access to and integration of health services within prisons is a potential way to combat intra- and extramural TB.

## References

[1] (2020). Global Tuberculosis Report.

[2] Ministério da Saúde (MS). Secretaria de Vigilância em Saúde. Sistema Nacional de Vigilância em Saúde (2020). Boletim epidemiológico: Tuberculose.

[3] Cords O, Martinez L, Warren JL, O'Marr JM, Walter KS, Cohen T (2021). Incidence and prevalence of tuberculosis in incarcerated populations: a systematic review and meta-analysis. Lancet Public Health.

[4] BRASIL. Departamento de doenças de condições crônicas e Infecções sexualmente transmissíveis (2021). Tuberculose: Populações vulneráveis.

[5] Ministério da Saúde (MS). Secretaria de Vigilância em Saúde. Sistema Nacional de Vigilância em Saúde (2019). Manual de Recomendações para o Controle da Tuberculose no Brasil.

[6] Paião DS, Lemos EF, Carbone AD, Sgarbi RV, AL Junior, da Silva FM (2016). Impact of mass-screening on tuberculosis incidence in a prospective cohort of Brazilian prisoners. BMC Infect Dis.

[7] DEPEN. Departamento Penitenciário Nacional. Ministério da Justiça e Segurança Pública (2019). Levantamento Nacional de Informações Penitenciárias.

[8] Walter KS, Martinez L, Arakaki-Sanchez D, Sequera VG, Estigarribia Sanabria G, Cohen T (2021). The escalating tuberculosis crisis in central and South American prisons. Lancet.

[9] Ministério da Saúde (MS). Secretaria de Vigilância em Saúde. Sistema Nacional de Vigilância em Saúde (2021). Boletim Epidemiológico. Tuberculose.

[10] QGIS (2021). GNU General Public License.

[11] GEO-PMSCS (2021). Geoprocessamento.

[12] Sacchi F, Praça RM, Tatara MB, Simonsen V, Ferrazoli L, Croda MG (2015). Prisons as Reservoir for Community Transmission of Tuberculosis, Brazil. Emerg Infect Dis.

[13] Carbone ASS, Sgarbi RVE, Lemos EF, Paião DSG, Simionatto S, Castro ARCM (2018). Estudo multicêntrico da prevalência de tuberculose e HIV na população carcerária do Estado do Mato Grosso do Sul. Com Ciências Saúde.

[14] Estevan AO, Oliveira SM, Croda J (2013). Active and latent tuberculosis in prisoners in the central-west region of Brazil. Rev Soc Bras Med Trop.

[15] Ministério da Saúde (MS). Secretaria de Vigilância em Saúde. Sistema Nacional de Vigilância em Saúde (2018). Boletim Epidemiológico: Tuberculose.

[16] CEVS. Centro Estadual de Vigilância em Saúde (RS) (2020). Programa Estadual de Controle da Tuberculose - PECT/RS.

[17] SUSEPE (2021). Superintendência dos serviços penitenciários do RS.

[18] Ministério da Saúde (MS). Secretaria de Vigilância em Saúde. Sistema Nacional de Vigilância em Saúde (2019). Boletim Epidemiológico: Tuberculose.

[19] Barbosa IR, Costa ICC (2014). Estudo epidemiológico da coinfecção Tuberculose-HIV no nordeste do Brasil. Rev Patol Trop.

[20] Corrêa APV, Feltrin AFS, Rodrigues IC, MAS Ponce, Santos MLSG, Vendramini SHF (2019). Aspectos associados ao desfecho do tratamento da coinfecção tuberculose/vírus da imunodeficiência humana. Enferm Bras.

[21] BRASIL (2014). Portaria Interministerial n. 1, de 02 de janeiro de 2014. Institui a Política Nacional de Atenção Integralà Saúde das Pessoas Privadas de Liberdade no Sistema Prisional (PNAISP) no âmbito do Sistema Único de Saúde (SUS).

[22] Allgayer MF, Ely KZ, Freitas GH, Valim ARM, Gonzales RIC, Krug SBF (2019). Tuberculosis: health care and surveillance in prisons. Rev Bras Enferm.

[23] Ely ZK, Dotta RM, Jarczewski CA, Valim ARM, Possuelo LG (2020). Diagnóstico bacteriológico de tuberculose na população privada de liberdade: ações desenvolvidas pelas equipes de atenção básica prisional. J Bras Pneumol.

